# Supplementary material for: Phase 3 Randomized, Multicenter, Placebo-Controlled Study to Evaluate Safety, Immunogenicity, and Lot-to-Lot Consistency of an Adjuvanted Cell Culture-Derived, H5N1 Subunit Influenza Virus Vaccine in Healthy Adult Subjects
Source: Vaccines (Basel). 2022 Mar 23;10(4):497. doi: 10.3390/vaccines10040497 (PMC9027673; doi:10.3390/vaccines10040497)
Supplement: Supplementary file 1 [file vaccines-10-00497-s001.zip › vaccines-1599014-supplementary.pdf]

Supplementary Materials

Phase 3 Randomized, Multicenter, Placebo-Controlled Study to Evaluate Safety, Immunogenicity, and Lot-to-Lot Consistency of an Adjuvanted Cell Culture-Derived, H5N1 Subunit Influenza Virus Vaccine in Healthy Adult Subjects

James Peterson, Esther Van Twuijver, Eve Versage and Matthew Hohenboken

Table S1. Immune responses (GMT and GMR) against aH5N1 on Days 1, 22, 43, and 183 by CHMP age group.[a.]

	**Age 18 to <60 years**		**Age ≥60 years**	
	**aH5N1c**	**Placebo**	**aH5N1c**	**Placebo**
Day 1, n	980	331	1269	408
GMT (95% CI)	13.0 (12.3–13.8)	13.4 (12.2–14.7)	20.1 (19.1–21.3)	20.1 (18.3–22.0)
Day 22, n	979	329	1266	407
GMT (95% CI)	51.1 (47.9–54.6)	11.2 (10.0–12.5)	42.9 (40.5–45.3)	14.4 (13.1–15.9)
GMR, Day 22/Day 1 (95% CI)	**3.92** (3.67–4.18)	0.85 (0.76–0.96)	**2.22** (2.10–2.35)	0.75 (0.68–0.82)
Day 43, n	947	309	1209	391
GMT (95% CI)	177.4 (166.2–189.4)	10.7 (9.5–11.9)	100.7 (95.0–106.7)	16.2 (14.7–17.9)
GMR, Day 43/Day 1 (95% CI)	**13.44** (12.59–14.35)	0.81 (0.72–0.90)	**5.18** (4.88–5.49)	0.83 (0.76–0.92)
Day 183, n	899	302	1180	385
GMT (95% CI)	20.4 (19.3–21.7)	6.7 (6.0–7.4)	19.3 (18.3–20.4)	8.5 (7.7–9.3)
GMR, Day 183/Day 1 (95% CI)	1.56 (1.47–1.66)	0.51 (0.46–0.57)	0.99 (0.94–1.05)	0.44 (0.40–0.48)

Abbreviations: CHMP, Committee for Medicinal Products for Human Use; CI, confidence interval; GMT, geometric mean titer; GMR, geometric mean ratio. [a] Boldface indicates CHMP criteria for GMR were met, i.e., GMR >2.5 for subjects aged 18 to <60 years and >2.0 for subjects aged ≥60 years.

Table S2. Percentages of subjects (95% CI) achieving seroconversion in HI titer.[a]

	**Age 18 to <60 years**		**Age ≥60 years**	
	**aH5N1c**	**Placebo**	**aH5N1c**	**Placebo**
Day 22, n	979	329	1266	407
Seroconversion, % (95% CI)	**42.2** (39.1–45.4)	1.5 (0.5–3.5)	24.6 (22.2–27.0)	0.7 (0.2–2.1)
Day 43, n	947	309	1209	391
Seroconversion, % (95% CI)	**81.6** (79.0–84.0)	0.3 (0.0–1.8)	**55.4** (52.6–58.2)	1.5 (0.6–3.3)
Day 183, n	899	302	1180	385
Seroconversion, % (95% CI)	17.0 (14.6–19.6)	0.3 (0.0–1.8)	8.2 (6.7–9.9)	1.0 (0.3–2.6)

Abbreviations: CHMP, Committee for Medicinal Products for Human Use; CI, confidence interval; HI, hemagglutination inhibition. [a] Seroconversion was defined as either a prevaccination (baseline) HI titer <1:10 and postvaccination HI titer ≥1:40 or a prevaccination HI titer ≥1:10 and a ≥4-fold increase in postvaccination HI antibody titer. Boldface indicates CHMP criteria for seroconversion were met, i.e., the percentage of subjects with seroconversion was ≥40% for subjects aged <60 years and ≥30% for subjects aged ≥60 years.
